# Biomarkers related to fatty acid oxidative capacity are predictive for continued weight loss in cachectic cancer patients

**DOI:** 10.1002/jcsm.12817

**Published:** 2021-10-11

**Authors:** Silvia Catanese, Carl Friedrich Beuchel, Teresa Sawall, Florian Lordick, Rommy Brauer, Markus Scholz, Uta Ceglarek, Ulrich T. Hacker

**Affiliations:** ^1^ Department of Oncology, Gastroenterology, Hepatology, Pulmonology and Infectious Diseases, University Cancer Center Leipzig (UCCL) Leipzig University Medical Center Leipzig Germany; ^2^ Department of Oncology University Hospital of Pisa Pisa Italy; ^3^ Institute for Medical Informatics, Statistics and Epidemiology (IMISE) Medical Faculty of the University Leipzig Leipzig Germany; ^4^ SRH Clinic Naumburg Naumburg Germany; ^5^ Institute of Laboratory Medicine, Clinical Chemistry and Molecular Diagnostics Leipzig University Medical Center Leipzig Germany

**Keywords:** Cancer, Cachexia, Metabolomics, Mass spectrometry, Biomarkers, Weight loss

## Abstract

**Background:**

Cachexia is characterized by a negative protein and energy balance leading to loss of adipose tissue and muscle mass. Cancer cachexia negatively impacts treatment tolerability and prognosis. Supportive interventions should be initiated as early as possible. Biomarkers for early prediction of continuing weight loss during the course of disease are currently lacking.

**Methods:**

In this pilot, observational, cross‐sectional, case–control study, cachectic cancer patients undergoing systemic first‐line cancer treatment were matched 2:1 with healthy controls according to age, gender and body mass index. Alterations in amino acid and energy metabolism, as indicated by acylcarnitine levels, were analysed using mass spectrometry in plasma samples (PS) and dried blood specimen (DBS). Welch's two‐sample *t*‐test was used for comparative analysis of metabolites between cancer patients and healthy matched controls and to identify the metabolomic profiles related to weight loss across different time points. A linear regression model was applied to correlate weight loss and single metabolites as predictor variables. Finally, metabolite pathway enrichment analyses were performed.

**Results:**

Eighteen cases (14 male and 4 female) and 36 paired controls were enrolled. There was a good correlation between baseline PS and DBS of healthy controls for the levels of most amino acids but not for acylcarnitine. Amino acid levels related to cancer metabolism were significantly altered in cancer patients compared with controls in both DBS and PS for arginine, citrulline, histidine and ornithine and in DBS only for asparagine, glutamine, methylhistidine, methionine, ornithine, serine, threonine and leucine/isoleucine. Metabolite enrichment analysis in PS of cancer patients revealed histidine metabolism activation (*P* = 0.0025). Baseline acylcarnitine analysis in DBS was indicative for alterations of the mitochondrial carnitine shuttle, related to β‐oxidation: The ratio palmitoylcarnitine/acylcarnitine (Q2) and the ratio palmitoylcarnitine + octadecenoylcarnitine/acylcarnitine (Q3) were predictive for early weight loss (*P* < 0.0001) and weight loss during follow‐up. Activation of tryptophan metabolism (*P* = 0.035) in DBS and PS and activation of serine/glycine metabolism (*P* = 0.017) in PS were also related to early weight loss and across successive time points.

**Conclusions:**

We found alterations in amino acid levels most likely attributable to cancer metabolism itself in cancer patients compared with controls. Baseline DBS represent a valuable analyte to study energy metabolism related to cancer cachexia. Acylcarnitine patterns (Q2, Q3) predicted further weight loss in cachectic cancer patients undergoing systemic therapy, and pathway analyses indicated involvement of the serine/glycine and the tryptophan pathway in this condition. Validation in larger cohorts is warranted.

## Introduction

Cachexia is a multifunctional metabolic syndrome characterized by involuntary weight loss due to wasting of skeletal muscle mass and/or adipose tissue degradation, affecting about 30–90% of cancer patients, preferentially at advanced tumour stages. Cachexia and particularly sarcopenia (i.e. decreased muscle mass and/or quality) negatively impacts efficacy and tolerability of different systemic cancer treatments (i.e. chemotherapy, molecular targeted therapy and immunotherapy) and causes functional impairments, further decreasing quality of life.^1^ Despite the fact that 10–20% of cancer related deaths are assumed to be associated with cachexia/sarcopenia, it still remains an underestimated and undertreated medical condition.^2^ Although international consensus has been achieved in defining clinical criteria as well as different stages of cachexia (i.e. pre‐cachexia, cachexia and refractory cachexia),^3^ robust biomarkers predicting progressive weight loss in cancer patients during the course of the disease are lacking. Such biomarkers, however, are urgently needed, because they could trigger early intensification of supportive measures (i.e. nutritional support) in patients at high risk for continuing weight loss. Moreover, such biomarkers could contribute to the identification of novel treatment targets.

Inflammatory cytokines like interleukin 6 (IL‐6) and tumour necrosis factor‐α (TNF‐α) released from tumour cells or the tumour microenvironment play a central pathophysiological role. They trigger acute phase response in the liver and alterations in metabolic processes related to amino acid and energy metabolism, leading to degradation of adipose tissue and muscle as well as neuroendocrine activation. Amino acids represent central intermediates in protein metabolism. Acylcarnitines, on the other hand, are transport forms of long‐chain fatty acyl‐CoA thioesters. They are formed in a reaction of L‐carnitine and acyl‐CoAs by carnitine acyltransferases located at the inner mitochondrial membrane to facilitate mitochondrial entry for β‐oxidation. Thus, they represent central intermediates of energy metabolism, which are in addition are indirectly involved in protein/amino acid metabolism.

Consequently, we aimed at analysing amino acids and acylcarnitine profiles in the blood as a promising approach for the identification of novel cancer cachexia‐related biomarkers.

Mass spectrometry (MS) is a well‐established method to simultaneously analyse multiple metabolic parameters from small blood samples. Importantly, metabolome analyses in the context of cancer cachexia were usually performed in serum, plasma or urine.^4^, ^5^ For example, Cala et al.^6^ used plasma samples from cachectic and non‐cachectic cancer patients to identify amino acids and their derivatives indicative for cachexia as well as a number of metabolite pathway alterations. Also, Yang et al.^4^ performed comparative metabolomics analyses in serum and urine samples to finally build a diagnostic model of cachexia based on three (i.e. carnosine, leucine and phenyl acetate) metabolites. DBS, on the other hand, reflects actual cellular metabolic processes and are currently used for the diagnosis of a number of inborn metabolic disorders for routine screening of newborns.^7^ DBS have already been used to identify novel metabolomic biomarkers to improve cancer diagnosis.^8^ DBS are simple to collect and can easily be stored and shipped, and MS‐based analysis in DBS has been extensively standardized during recent years.^9^ In addition, PS and DBS can in part be considered complementary as alterations in energy metabolism—like acylcarnitine profiles—can be detected in DBS with higher sensitivity.

From this background, in our pilot cross‐sectional, case–control study, we choose to perform MS‐based analysis of blood amino acid and acylcarnitine profiles both in DBS and PS from cachectic patients with advanced gastrointestinal cancers. In PS, we found alterations of plasma amino acid levels related to the urea cycle and histidine pathway activation in cachectic cancer patients compared with matched healthy controls. Pathway analyses in PS indicated involvement of the serine/glycine and the tryptophan pathway in patients with continuous weight loss. More importantly, in DBS, we were able to identify a characteristic acylcarnitine pattern at baseline, which significantly predicted further weight loss.

## Methods

### Patient selection and sample collection

From October 2014 to January 2016, 19 cancer patients (patient No. 13 was excluded later because of the absence of neoplastic disease) were consecutively enrolled in a pilot, observational, cross‐sectional, case–control study, at the University Cancer Centre Leipzig (UCCL) central outpatient unit according to the following inclusion criteria: age ≥18 years, newly diagnosed, histologically confirmed gastrointestinal malignancy, planned to undergo systemic chemotherapy treatment, meeting the definition of cachexia, according to the Consensus Conference definition.^3^ Patients were excluded in case of active infections, uncontrolled diabetes, kidney or liver failure or immunodeficiency syndromes. Cancer patients consecutively enrolled in our study were 1:2 matched by age, sex and body mass index with healthy controls (*n* = 36), from the population‐based LIFE‐Adult study (https://life.uni‐leipzig.de/en/life_health_study.html; accessed 20 July 2020).^10^ Only healthy subjects were selected based on an extensive questionnaire covering a broad range of diseases (i.e. prior or present cancer diagnosis, cardiovascular diseases, lung diseases, gastrointestinal/liver diseases, renal diseases, autoimmune diseases, metabolic/endocrine diseases, musculoskeletal diseases, neurological diseases, eye diseases, dermatological disease, allergies, infectious diseases and psychiatric conditions including depression). Moreover, a panel of laboratory parameters was available for every participant in the LIFE study including the following parameters related to metabolism and inflammation: C‐reactive protein (CRP) ≤ 5 mg/L; interleukin 6 (IL‐6) < 7 pg/mL; thyroid‐stimulating hormone (TSH) 0.4–2.5 mU; glucose 3.9–6.1 mmol/L (70–110 mg/dL); haemoglobin A1c (HbA1c) 20–42 mmol/mol bzw. 3–6%; triglyceride < 1.7 mmol/L; cholesterol: women 5.1–7.2 mmol/L, men 4.5–6.2 mmol/L; high‐density lipoprotein (HDL)‐cholesterol > 1.03 mmol/L; low‐density lipoprotein (LDL‐cholesterol) < 4.2 mmol/L. In the controls selected for our study, all parameters were in the normal range.

Blood samples were collected from all patients/controls in the morning after a fasting period of around eight to 14 h. Thereafter, blood samples of patients were collected every 4 weeks (±7 days) for up to six consecutive time periods: Time Point 1 (TP1) represents baseline visit in the study, TP2 to TP7 represent respective follow‐up visits (*Figure* *S1*). EDTA–plasma samples were immediately centrifuged for 10 min at 2750 g at 15°C and stored within 2 h of collection at −80°C until analysis. Weight developments were closely monitored. This study was conducted under the approval of the Ethics Committee of the Medical Faculty of the University Leipzig (AZ:137/14‐ff) and in accordance with the principles of the Declaration of Helsinki. Before enrolment, all patients provided written informed consent.

### Metabolomics measurements

Mass spectrometric analysis of amino acids and acylcarnitines was performed on EDTA–plasma (PS) and dried EDTA–whole blood samples (DBS) using an API 2000 tandem mass spectrometer (Applied Biosystems, Germany) and a Turbo Ion Spray Source (TIS) in combination with a HTC Pal autosampler and a PE 200 microgradient pump for flow injection analysis (FIA). The methodology has been described in detail elsewhere.^9^ Data related to the cancer patient cohort are provided on Zenodo at: https://doi.org/10.5281/zenodo.5122502.

### Statistical analysis

Metabolite data were filtered for outliers using a cut‐off of mean +5 × SD of the logarithmized data (no measurements had to be removed as outliers). Afterwards, data were inverse‐normal‐transformed to ensure normal distribution of the measurements while retaining measurements at zero. Thus, metabolites were analysed as standardized measurements with a mean μ = 0 and a standard deviation SD = 1. Correlation between baseline PS and DBS metabolome of cases plus controls and of controls only was calculated as Spearman's rho using the cor.test() function of the ‘stats’ R package Version 3.6.0, including a multiple testing correction with a false discovery rate (FDR) = 5% after performing the Benjamini–Hochberg procedure. In addition, correlation of baseline IL‐6 PS levels and baseline metabolite levels both in DBS and PS were analysed accordingly.

Differences in metabolite levels both in PS and DBS between cancer patients and healthy matched controls were analysed using Welch's two‐sample *t*‐test as implemented in the t.test()function of the ‘stats’ R package including correction for multiple comparisons as indicated by *q*‐values, computed using the p.adjust() function using the parameter “method = ‘BH’”.

For testing metabolite associations with weight loss within the cases, metabolite data for controls were averaged for each pair and subtracted from its corresponding case for each respective time point. The weight loss phenotype was defined as the difference of the weight from a given time point and the successive time point. The following weight loss definitions are used: ‘initial weight loss’, calculated as the difference of the weight from baseline (TP1) and the long‐term historical weight based on anamnestic information from the patients, and ‘early weight loss’, indicating weight loss between baseline (TP1) and the subsequent TP2.

The binary variable (weight loss: 0 = no/1 = yes) was defined as a weight loss of more than 2% compared with the previous time point. Association of single metabolites with the binary weight loss phenotype from TP1 to TP2 was tested using Welch's two‐sample *t*‐test. Metabolites nominally (at *P* < 0.05) associated with the binary weight loss phenotype from TP1 to TP2 were subsequently also tested for association with binary weight loss separately at the remaining available time points in a pairwise comparison of successive time points (up to TP7). Associations of single metabolites with weight loss in kg from TP1 to TP2 were tested using linear regression models as implemented in the ‘lm()’ function of the ‘stats’ R package with the weight loss as response variable and each metabolite as singular predictor variable. All *P*‐values were adjusted for multiple testing controlling the FDR at 5%. Due to the limited sample size, multivariable analysis of correlated analytes was omitted. Finally, pathway enrichment was tested using MetaboAnalyst 4.0's MetPa tool.^11^, ^12^ For significantly associating metabolites (at FDR = 5%), we performed a pathway analysis of KEGG‐metabolic pathways using all representable metabolites (M = 32) as background.^13^ All statistical analyses were performed using the open‐source statistical software package R 3.6.0^14^ (https://www.R‐project.org).

## Results

### Patient characteristics

Characteristics of the 18 cachectic cancer patients enrolled are given in *Table* *S1*. 78% of the patients were male. All but one patient who received chemotherapy in an adjuvant setting were treated for advanced or metastatic gastrointestinal cancers (i.e. mostly gastro‐oesophageal cancer in 67% of cases; *Table* *S1*). Median age was 61 years (range 51–79). Though all patients were classified as cachectic at study inclusion, with a weight loss >5% in the past 6 months in comparison with the historical weight or weight loss >2% in the past 6 months and a BMI < 20 kg/m^2^, according to the consensus definitions,^3^ *n* = 8 of them presented with a normal weight BMI category, and none of them were underweight.

Weight trends were assessable in 14 of the 18 cancer patients enrolled according to the definition (see Methods section) and five patients presented with weight loss from baseline (TP1) to TP2, whereas seven patients showed weight loss >2% within at least one interval of consecutive time points during the entire time course (*Figure* *S1*).

### Comparison of metabolomics analyses in PS vs. DBS in healthy controls

Because we performed our metabolomics analysis in parallel in PS and DBS, we were interested in the correlation of metabolite levels between these two specimens. For this purpose, we analysed the data set of healthy controls. Distribution of transformed data are shown in *Figure* *S2* *A*, and significant correlations are given in *Table* *1* (see *Table* *S2* for the rest of the data set). The following metabolites or metabolite ratios showed very strong or strong correlation levels (ϱ ≥ 0.6): proline, alanine, phenylalanine, threonine, glycine and citrulline and free carnitine, decanoylcarnitine, the ratio Q11 (i.e. alanine/acetylcarnitine) and finally aminobutyric acid.

**Table 1:** Spearman correlation in healthy controls between metabolite in DBS and PS and of distinct ratios of metabolites

Metabolite	Spearman ϱ	*P*‐value	*q*‐Value
Proline	0.86	<0.0001	<0.0001
Alanine	0.81	<0.0001	<0.0001
Aminobutyric acid	0.74	<0.0001	<0.0001
Phenylalanine	0.72	<0.0001	<0.0001
Threonine	0.72	<0.0001	<0.0001
Carnitine free	0.65	<0.0001	0.0002
Q11: alanine/acetylcarnitine	0.66	<0.0001	0.0002
Decanoylcarnitine	0.65	<0.0001	0.0002
Glycine	0.64	<0.0001	0.0002
Citrulline	0.60	0.0001	0.0007
Leucine/isoleucine	0.57	0.0003	0.0019
Tyrosine	0.56	0.0004	0.0025
Sarcosine	0.53	0.0009	0.0052
Methylhistidine	0.52	0.0012	0.0065
Butyrylcarnitine	0.50	0.0020	0.0097
Arginine	0.46	0.0043	0.0195
Acetylcarnitine	0.46	0.0052	0.0224
Q13: arginine/citrulline	0.44	0.0073	0.0296
Serine	0.43	0.0081	0.0310
Q6: glutarylcarnitine/lysine	0.43	0.0092	0.0334
Hydroxyproline	0.40	0.0146	0.0500
Pipecolic acid	0.40	0.0151	0.0500

See Burkhardt et al.^15^ and *Table* *S5*.

According to spearman rho (ϱ) value, the entity of correlations is defined as follows: very strong if ϱ ≥ 0.8; strong ϱ 0.6–0.79; moderate ϱ 0.40–0.59; low ϱ 0.20–0.39; ϱ < 0.20 no correlation. Only correlations with a *q*‐value ≤ 0.05 following correlation for multiple comparisons are included in the table (full data set, see *Table* *S2*).

### Comparison of metabolomics analyses in PS and DBS in healthy controls vs. cancer patients

Next, we compared metabolite levels in PS and DBS between cancer patients and healthy controls. Values of arginine, citrulline and histidine were significantly reduced in both DBS and PS in cachectic cancer patients compared with healthy controls (*Table* *2*). Moreover, values of asparagine, glutamine, methyl‐histidine, methionine, ornithine, serine and threonine as well as the ratio of the branched‐chain amino acids (BCAA) leucine/isoleucine were significantly reduced in DBS but not in PS of cancer patients compared with controls (*Table* *2*). In contrast, ornithine levels were significantly increased in PS of cancer patients compared with controls. Finally, no clear pattern could be observed in this comparison with respect to the acylcarnitine measurements: Whereas hexanoylcarnitine (C6) was significantly decreased in DBS and hexadecenoylcarnitine (C16:1) and octenoylcarnitine (C8:1) in PS of the cancer patients compared with controls, free carnitine was significantly increased in the plasma (*Table* *2*) (*Table* *S3* for the remaining set of data and *Figure* *S2* for the entire data set).

**Table 2:** Comparison of estimated mean differences between patients and controls of standardized measurements of amino acids, carnitine, free carnitine, acylcarnitines and free fatty acids in DBS and their corresponding values in PS

Metabolite	DBS			Plasma		
	Mean difference	95% CI	*q*‐Value	Mean difference	95% CI	*q*‐Value
Arginine	−1.53	−2 to −1.07	0.0001	−1.31	−1.66 to −0.955	<0.0001
Asparagine	−0.803	−1.19 to −0.419	0.0043	0.258	−0.307–0.824	0.5788
C16:1 hexadecenoylcarnitine	0.135	−0.543–0.814	0.8118	−0.961	−1.5 to −0.427	0.0150
C6: hexanoylcarnitine	−0.917	−1.57 to −0.268	0.0448	0.105	−0.501–0.711	0.8891
C8:1 octenoylcarnitine	−0.389	−0.932–0.154	0.3465	−0.916	−1.51 to −0.325	0.0410
Carnitine free	−0.703	−1.47–0.0627	0.1876	1.05	0.573–1.53	0.0029
Citrulline	−1.37	−1.81 to −0.932	0.0001	−1.12	−1.57 to −0.663	0.0011
Glutamine	−0.91	−1.55 to −0.273	0.0448	−0.287	−0.987–0.413	0.6060
Histidine	−1.84	−2.25 to −1.44	>0.0001	−1.45	−1.84 to −1.05	>0.0001
Leucine/isoleucine	−0.676	−1.13 to −0.221	0.0420	−0.414	−1–0.173	0.4740
Methylhistidine	−1.09	−1.6 to −0.574	0.0043	−0.697	−1.3 to −0.0985	0.1664
Methionine	0.889	0.274–1.5	0.0448	0.365	−0.286–1.02	0.4907
Ornithine	−1.61	−1.99 to −1.22	>0.0001	1.14	0.694–1.59	0.0009
Q14: arginine/ornithine	−0.41	−1.04–0.218	0.3997	−1.36	−1.71 to −1.01	>0.0001
Q18: triglylcarnitine/leucine/isoleucine	0.725	0.293–1.16	0.0214	0.315	−0.265–0.895	0.4907
Serine	−1.24	−1.79 – −0.677	0.0036	−0.283	−0.862–0.297	0.5517
Threonine	−1.07	−1.67 – −0.472	0.0149	−0.579	−1.25–0.0892	0.3885

Negative values correspond to an overall lower mean in the patient group compared with the control group, and positive values to a higher mean. Only significant differences in either DBS or PS (with a *q*‐value ≤ 0.05 following correlation for multiple comparisons) are reported. The remaining set of data is given in *Table* *S3*.

95% CI, 95% confidence interval; DBS, dried blood sample.

Because systemic inflammation represents a key pathophysiological mechanism in propagating development of cancer‐related cachexia, we performed an additional analysis to identify potential correlations between IL‐6 levels and the metabolomic parameters. We found no statistically significant correlation between baseline IL‐6 levels and any of the metabolomic parameters studied, neither in PS nor in DBS (data not shown).

Next, pathway enrichment analysis (based on data from PS) indicated that cancer cachexia triggers histidine metabolism reprogramming (*P* = 0.0025), according to MetaboAnalyst's MetPa tool, which is related to alanine, aspartate and glutamate metabolism, the purine metabolism and the pentose phosphate pathway.

### Predictive metabolomics model of weight loss in cachectic cancer patients

To identify potential biomarkers related to weight loss, we performed linear regression analysis with weight loss in kg as response and metabolite levels as predictor variables. First, we focused on initial weight loss (i.e. loss from historic weight to the weight at baseline [TP1]). Whereas some parameters both from baseline amino acid and acylcarnitine analyses (i.e. hydroxyproline, ornithine, tryptophan and leucine/isoleucine as well as C14 and C20:2) reached statistical significance in the primary analysis (*P* < 0.05), none of them retained statistical significance after correction for multiple testing (*n* = 74 analytes) (*Table* *S4* *A*).

Next, based on univariate linear regression analysis with weight loss in kg as response and metabolite levels as predictor variables, we searched for biomarkers related to early weight loss (i.e. between baseline [TP1] and TP2) and identified significantly (*P* < 0.05) decreased baseline levels of alanine (in DBS and PS) as well as baseline plasma ornithine and sarcosine levels. Regarding acylcarnitine, C8:1 (in PS), C10 and C18 (in DBS), C16OH in PS as well as C20:1 and the ratios Q2 (palmitoylcarnitine/acetylcarnitine) and Q3 (palmitoylcarnitine + octadecenoylcarnitine/acetylcarnitine) in DBS were altered with nominal significance in these patients. Following correction for multiple testing (*n* = 74 analytes), significance was no longer reached; however, a trend remained for C20:1 and the ratios Q2 (C16/C2) and Q3 (C16 + C18:1/C2), *P* = 0.1499, respectively (*Table* *S4* *B*).

Next, we compared baseline amino acid and acylcarnitine levels between cachectic patients with stable weight and those with weight loss (i.e. change of ≥2% between each time point and the subsequent one using the categories weight loss: yes/no) both in DBS and in PS.

Using Welch's two‐sample *t*‐test and these binary weight loss categories, according to our definition, Q2 and Q3 (in DBS) turned out to be predictive (*P* = 0.00013 and *P* < 0.0001, respectively) for further weight loss between baseline (TP1) and the first control time point (TP2), thus indicating early weight loss. These parameters maintained statistical significance after correction for multiple testing (including all *n* = 74 analytes), *P* = 0.017 for both (*Table* *3*).

**Table 3:** Metabolome differences between cachectic patients with weight loss (WL) vs. stable weight (WS)

		Estimated mean value WS/WL^a^	95% CI of mean value diff.	*P*‐value	*q*‐Value
Alanine	DBS	0.379/−0.715	0.163–2.02	0.0252	0.3688
Alanine	Plasma	0.378/−0.562	0.173–1.71	0.0204	0.5137
Tryptophan	Plasma	0.593/−0.625	0.0819–2.36	0.0378	0.5137
3‐Methylglutaylcarnitine	Plasma	0.921/−0.438	0.00536–2.71	0.0493	0.5137
Ornithine	Plasma	1.44/0.607	0.0488–1.63	0.0392	0.5137
C16OH	Plasma	0.0485/1.05	−1.91 to −0.0897	0.0347	0.5137
C18	Plasma	0.933/−0.675	0.0683–3.15	0.0430	0.5137
C201	DBS	−0.924/0.479	−2.32 to −0.49	0.0064	0.1559
C6DC	Plasma	0.922/−0.453	0.0286–2.72	0.0461	0.5137
Q14	DBS	−1.06/0.288	−2.27 to −0.417	0.0097	0.1767
Q2	DBS	−0.404/1.37	−2.66 to −0.879	0.0013	0.0459
Q3	DBS	−0.0879/1.6	−2.45 to −0.919	0.0007	0.0459

Results are given according to binary categorization of weight loss from baseline (TP1) to the second (TP2) scheduled evaluation (Welch's two‐sided *t*‐test), *n* = 14. Only results with *P*‐value < 0.05 are reported. *q*‐Values represent results following correction for multiple testing.

95% CI, 95% C.I., 95% confidence interval; diff., difference; WL, weight loss; WS, weight stable.

^a^ Values represent SD.

Moreover, we tested for weight loss between all successive time points (i.e. pairwise comparison between the remaining available successive time points) in metabolites, reaching at least nominal significance for weight loss between TP1 and TP2. Again, Q2 and Q3 (in DBS) were shown to be the only remaining predictive parameters, following correction for multiple testing, *P* = 0.0459 for both (*Table* *4*).

**Table 4:** Metabolite differences between cachectic cancer patients with weight loss (WL) vs. stable weight (WS)

		Estimated mean value WS/WL	95% CI of mean value diff.	*P*‐value	*q*‐Value
Alanine	DBS	0.379/−0.715	0.163–2.02	0.0253	0.0974
Alanine	Plasma	0.378/−0.562	0.173–1.71	0.0205	0.0965
Ornithine	Plasma	1.44/0.607	0.0488–1.63	0.0392	0.1177
Sarcosine	Plasma	0.939/−1.5	0.589–4.28	0.0215	0.0965
C16OH	Plasma	0.0485/1.05	−1.91 to −0.0897	0.0347	0.1170
C201	DBS	−0.924/0.479	−2.32 to −0.49	0.0064	0.0577
C81	Plasma	−0.35/−2.35	0.608–3.4	0.0156	0.0965
Q2	DBS	−0.404/1.37	−2.66 to −0.879	0.0013	0.0170
Q3	DBS	−0.0879/1.6	−2.45 to −0.919	0.0007	0.0170

Results are given according to binary categorization of weight loss for all time points (Welch's two‐sided *t*‐test), *n* = 14. Only results with *P*‐value < 0.05 are reported. *q*‐Values represent results following correction for multiple testing.

95% CI, 95% confidence interval; diff., difference; WL, weight loss; WS, weight stable.

Pathway metabolite enrichment analysis indicated alterations in serine/glycine metabolism in PS of cachectic patients with continued weight loss (*P* = 0.017, according to MetaboAnalyst's MetPa tool). In addition, pathway plasma metabolite enrichment analysis indicated an altered tryptophan metabolism network (*P*= 0.0347, according to MetaboAnalyst's MetPa tool) in both PS and DBS in cachectic cancer patients with early weight loss between baseline (TP1) and the first control time point (TP2).

## Discussion

Early multimodal interventions (i.e. nutritional support, exercise training and anti‐inflammatory interventions) have been suggested for the treatment of cancer‐related cachexia. Consequently, clinical recognition of malnutrition and cachexia is important.^2^ Thus, there is an urgent need to identify biomarkers predicting continued weight loss in cachectic cancer patients. Such markers could both trigger early intensification of supportive interventions and provide insights into the pathophysiology of this phenomenon, which could help to identify potential novel treatment targets.

This prospective case–control study relies on tandem MS‐based analysis of DBS and PS in a cohort of cachectic gastrointestinal cancer patients and matched healthy controls to identify markers predictive for further weight loss during first‐line cancer treatment. Loss of muscle mass (i.e. sarcopenia) is a well‐defined negative prognostic marker in cancer patients. Pathophysiologically, fatty acid and muscle metabolism are interrelated in triggering muscle loss.^16^ Consequently, we choose to analyse both amino acid (i.e. related to protein/muscle metabolism) and acylcarnitine (i.e. related to fatty acid/energy metabolism) metabolism profiles. Such analyses have traditionally been performed in PS. Here, we introduced analysis in DBS and performed analyses in parallel in both analytical compartments. Specifically, whereas plasma has been used for amino acid analysis for decades, acylcarnitine can be measured by MS in DBS with high accuracy and sensitivity, which led to the widespread use of MS‐based analysis of DBS for routine newborn screening to identify rare inborn metabolic defects covering a wide spectrum from urea cycle, amino acid, organic acid and fatty acid metabolic disorders including CPT 1 and CPT 2 deficiencies.^17^

We found high correlations for a number of amino acids between PS and DBS although specifically long‐chain acylcarnitine levels did not correlate (*Tables* *1* and *S2*). As acylcarnitines play a central role in energy metabolism and are generated at the inner mitochondrial membrane of cells, the cellular content in DBS (mostly leucocytes) vs. PS may represent a possible explanation for these findings exclusively made in DBS analysis. Several studies indeed indicate that amino acid levels in plasma and DBS are highly correlated. For certain amino acids, however, lower concentrations have been found in DBS compared with plasma, and although the reasons for this bias are not fully understood, lower extraction efficiency may play a role.^18^, ^19^ For acylcarnitines, differences have also been described between plasma and DBS, and this mostly accounts for long‐chain acylcarnitines that are endogenously present in normal erythrocytes^20^ or can be absorbed in red blood cells.^21^

Comparing our cohort of cachectic cancer patients with matched controls, the following alterations in the levels of different amino acids in cancer patients were identified: Arginine, citrulline and histidine were significantly reduced in both DBS and PS of cachectic cancer patients. Moreover, asparagine, glutamine, leucine/isoleucine, methylhistidine, methionine, ornithine, serine and threonine were reduced in DBS (for full overview of differences, see *Tables* *2* and *S3*). Interestingly, ornithine levels were significantly decreased in DBS but significantly increased in PS. This may again reflect the contribution of cellular components in DBS. In this respect, increased levels of ornithine‐decarboxylase activity have been described in peripheral blood leucocytes from patients with chronic lymphocytic leukaemia.^22^ On the other hand, and in line with our findings, arginine levels were found decreased in the plasma of cancer patients, and conversion of arginine to ornithine by arginase was proposed as a mechanism.^23^ Interestingly, we also found a decreased ratio of arginine/ornithine in PS, possibly related to an increase in arginase activity in the tumour microenvironment.^24^ With respect to arginine, decreased levels are often found in malignant tumours, and conversion of arginine to ornithine by arginase produces polyamines (putrescine, spermine and spermidine) that promote tumour proliferation and aggressiveness through modulation of the global chromatin structure.^25^ Moreover, depletion of arginine contributes to suppression of cytotoxic T‐cell proliferation.^26^ In line with our findings, in a cohort of cachectic cancer patients (breast, colorectal and pancreatic cancer) with different levels of weight loss, decreased plasma free arginine concentrations were found to be tumour related, independently from weight loss.^23^ The ratio of essential BCAA leucine/isoleucine was reduced in our cohort of cancer patients. BCAA constitute a high‐level source of acetyl‐CoA sustaining the Krebs cycle or lipogenesis. Moreover, acetyl‐CoA is required for histone and protein acetylation, providing a link to epigenetic modifications and tumour growth.^27^ Moreover, enzymes catalysing the first step of BCAA catabolism are overexpressed in many cancers,^28^ again underscoring their role in cancer cell metabolism. Finally, histidine and methylhistidine were significantly decreased in both DBS and in PS of our cachectic cancer patients. It has recently been demonstrated that histidine levels correlate to some extent with the total amount of pro‐inflammatory cytokines.^29^ In our cohort, however, there was no correlation with IL‐6 levels. This discrepancy might be explained by the marked differences in the clinical characteristics of the cohort analysed by Sirnio et al. (i.e. mostly colorectal cancer patients, 13.1% Stage IV only) compared with our cohort. We also observed a global activation of the histidine metabolic pathway, according to PS measurements, in cachectic cancer patients compared with healthy controls (*P* = 0.0025). According of the metabolite enrichment analysis, this pathway is associated with the pentose phosphate pathway, alanine, aspartate and glutamine metabolism and thus with purine metabolism including all amino acids found to be significantly altered in our cancer cohort. In line with our findings, Cala et al.^6^ recently reported a decrease of histidine derivatives in cachectic cancer patients. The cancer patients included in our study were all defined by a weight loss >5% at baseline to define cachexia according to the consensus guidelines. It was the predominant criterion defining cachexia, according to the consensus criteria.^3^ None of them had a BMI < 20 kg/m^2^. Accordingly, none of the controls were underweight (i.e. BMI category < 18.5 kg/m^2^). Due to this fact and due to the lack of a group of cancer patients without cachexia according to the consensus criteria, it is not possible to delineate a specific role of amino acid biomarker alterations with respect to cachexia independently from cancer‐related alterations in a strict sense. In addition, our limited sample size does not allow for a multivariable analysis of correlated metabolites, which would be warranted when analysing closely related analytes. Overall, however, there is a broad body of evidence from the literature that amino acid metabolomic profiles in PS or DBS are dominantly influenced by cancer cell metabolism itself, thus restricting the value of single amino acids as specific cancer cachexia markers.

In line with this hypothesis, alterations in single amino acid levels were not predictive for further weight loss in our cohort. However, pathway analysis based on data derived from PS indicated activation of the glycine/serine pathway related to weight loss for successive time points (*P* = 0.017) as well as tryptophan pathway activation (*P* = 0.035) related to early (i.e. between TP1 and TP2) weight loss. Serine and glycine are related to the one carbon metabolism in cancer cells^30^ and were shown to be involved in cell transformation and malignancy.^31^ Moreover, it has been demonstrated recently that glycine administration attenuated muscle wasting in a mouse model of cancer cachexia.^32^

Tryptophan plays a pivotal role during T‐cell activation, and indoleamine‐(2,3)‐dioxygenase (IDO), which is induced by interferon‐γ (IFN‐γ), mediates degradation of tryptophan. In addition, a relation of tryptophan metabolism with inflammation is well established.^33^ In cachectic patients suffering from haematological malignancies, weight loss was associated with immune activation (i.e. IFN‐γ activity) as well as decreased serum tryptophan levels.^34^ Similarly, Iwagaki et al.^35^ demonstrated that decreased serum tryptophan levels in cachectic gastrointestinal cancer patients compared with controls and reduced nutritional state and further weight loss were associated with increased levels of neopterin and a consumption of tryptophan. From this background, IDO inhibitors, which are currently been developed as cancer drugs for immunotherapy,^36^ might also be interesting for their potential effects in cancer cachexia. Interestingly, in a CT‐26 murine tumour model, inhibition of IDO decreased tumour growth kinetics and efficiently prevented loss of body weight.^37^

With respect to energy metabolism‐related metabolomic markers, we could demonstrate that both Q2 and Q3 ratio in DBS of cachectic cancer patients predicted further weight loss according to binary categories (yes/no) between baseline evaluation (TP1) and TP2 during treatment. Q2 and Q3 comprise ratios between long‐chain acylcarnitine in the numerator, palmitoylcarnitine (C16) for Q2 and palmitoylcarnitine plus octadecenoylcarnitine (C16 + C18:1) for Q3 and acetylcarnitine (C2) in the denominator (for both Q2 and Q3). They reflect the activity of mitochondrial carnitine–palmitoyltransferase 1 and 2, corresponding to carnitine shuttle (CS) activity. CS plays a pivotal role in fatty acid metabolism, allowing the transport of long‐chain fatty acids across the impermeable mitochondrial membranes. These are usually esterified in the cytosol by coenzyme A (CoA) to form acyl‐CoA thioesters before beta‐oxidation in mitochondria. CS comprises four enzymes, namely, carnitine‐palmitoyltransferases I (CPT I) and II (CPT II), the carnitine acetyltransferase (CrAT) and the carnitine‐acylcarnitine translocase (CACT), which are involved in the bidirectional transport of acyl‐CoA and carnitine in exchange with CoA and acylcarnitine from the cytosol to mitochondria.^38^ In body fluids (i.e. PS and DBS), acylcarnitine profiles are not only a diagnostic test for inherited disorders of fatty acid metabolism but also for defects in BCAA catabolism. Specifically, elevation of Q2 or Q3, as seen in our cachectic cancer patient cohort loosing further weight compared with cachectic cancer patients maintaining or even gaining weight, represents an established marker for defective CPT II function^39^ in the clinical setting for newborn screening, which results in impaired β‐oxidation and/or liver ketogenesis.^21^ Interestingly, back in 1998, Seelaender et al.^40^ using a model of Walker 256 carcinosarcoma bearing rats affected by severe cachexia found a marked reduction (i.e. −56%) of activity of the isolated mitochondrial inner‐membrane CPT II in comparison with non‐tumour‐bearing, non‐cachectic control animals and identified a lower weight, catalytically less active isoform of CPT II, possibly induced by tumour‐derived factors (i.e. TNF‐α). Later on, the same group showed reduction of CPT II activity in hepatic sinusoids, possibly contributing to the progression of cachexia.^41^ In line with these findings, in a cachexia mouse model of colon‐26 adenocarcinoma, Liu et al. reported decreased mRNA expression levels and enzymatic activity of CPT I and CPT II and significantly reduced amounts of free carnitine and acetyl‐carnitine compared to normal controls^42^ and oral administration of high doses of L‐carnitine in this murine model^42^ or a rat model of cachexia^43^ improved the condition by reducing serum levels of IL‐6 and TNF‐α and restoring CPT expression and activity to enhanced beta‐oxidation and to decrease adipose tissue breakdown.^42^ As the predictive alterations in Q2 and Q3 were only found in DBS, it might be speculated that the metabolomic changes related to the CS described in the liver in experimental models so far^40^, ^42^ are reflected similarly in the cellular compartment (i.e. predominantly leucocytes) of DBS.

In summary, our results indicate that changes in acylcarnitine profiles measured in DBS and related to the CS are predictive for further weight loss in cachectic cancer patients. Moreover, pathway analyses indicated an involvement of the serine/glycine and the tryptophan pathway. Due to the small sample size, the findings are hypothesis generating and should be validated in larger cohorts.

## Conflicts of interest

All authors declare that they have no conflict of interest. The authors certify that they comply with the ethical guidelines for publishing in the *Journal of Cachexia, Sarcopenia and Muscle*: update 2019.^44^

## Supporting information


**Figure S1.** Single patient's weight variations over all time points. All patients were consecutively enrolled. Pat. 13 was excluded after study enrolment, because of absence of tumor tissue in the biopsy samples. Numbers on the x‐axis represent time points of measurements ranging from 1 (baseline) up to 7

**Figure S2.** A) Comparison of control standardized metabolite measurements in DBS and PS; B) Comparison of case–control differences among pre‐processed metabolite measurements in plasma and dried blood

**Table S1.** Baseline clinical characteristics and weight variations. Abbreviations: UICC, Union international contre le Cancer; BMI, Body Mass Index

**Table S2.** Spearman Correlation between metabolites in DBS and PS determined in the control cohort

**Table S3.** Comparison of estimated mean differences of standardized measurements between patients and controls of amino acids, carnitine, free carnitine, acyl‐carnitines and free fatty acids in PS and corresponding DBS. Negative values correspond to an overall lower mean in the patient group compared to the control group, positive values to a higher mean. A q value<0.05 indicates statistical significance following correction for multiple testing

**Table S4.** A) Linear regression analysis of metabolome profile predictive for initial weight loss as a continuous variable (according to numeric weight loss from the historical weight to the weight at TP1); B) Linear regression analysis of metabolome profile (according to numeric weight loss) from baseline to the second scheduled evaluation (i.e., early weight loss). Only results with p‐value <0.05 are reported, q‐values represent results following correction for multiple testing

**Table S5.** Complete overview of metabolites and metabolite ratios analyzed and mapped in the reference metabolite database as published by Burkhardt et al. [15]. HMDB and PubChem identifiers are indicated
