# Supplementary figures and images for: Biomarkers related to fatty acid oxidative capacity are predictive for continued weight loss in cachectic cancer patients
Source: J Cachexia Sarcopenia Muscle. 2021 Oct 11;12(6):2101–10. doi: 10.1002/jcsm.12817 (PMC8718041; doi:10.1002/jcsm.12817)

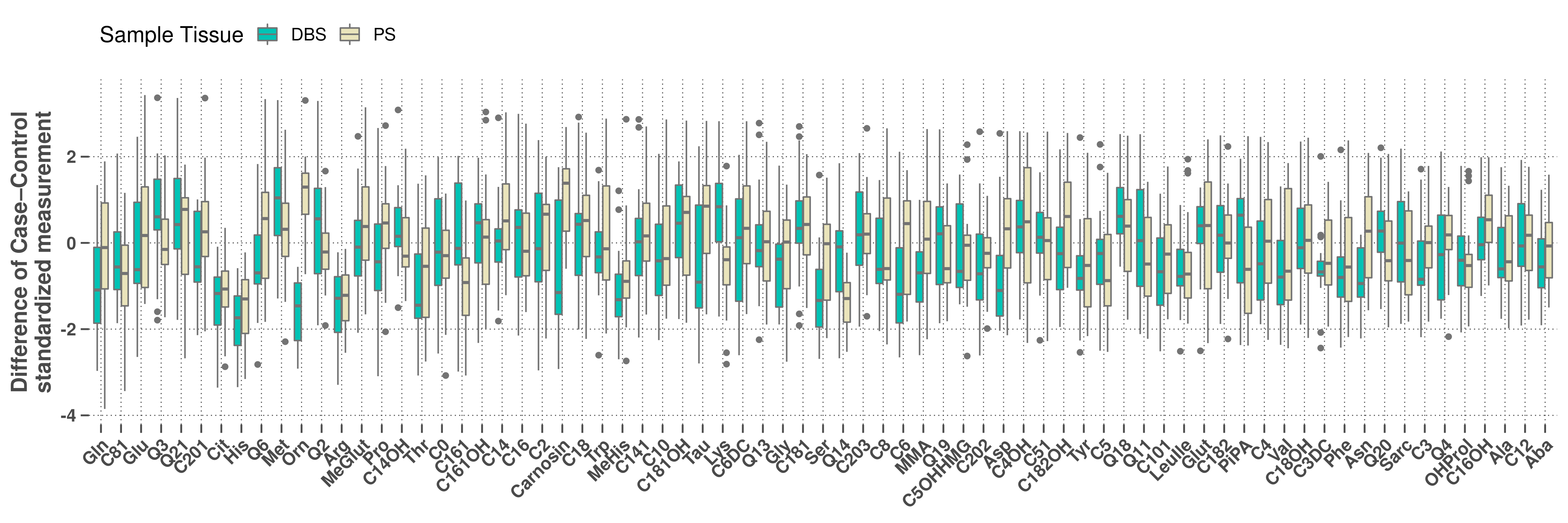

Supplement: Supplementary file 2 — Figure S2. A) Comparison of control standardized metabolite measurements in DBS and PS; B) Comparison of case–control differences among pre‐processed metabolite measurements in plasma and dried blood [file JCSM-12-2101-s005.zip › JCSM_12817_Supplemental Figure S2 A Correlations DBS vs. PS.revised.tiff]

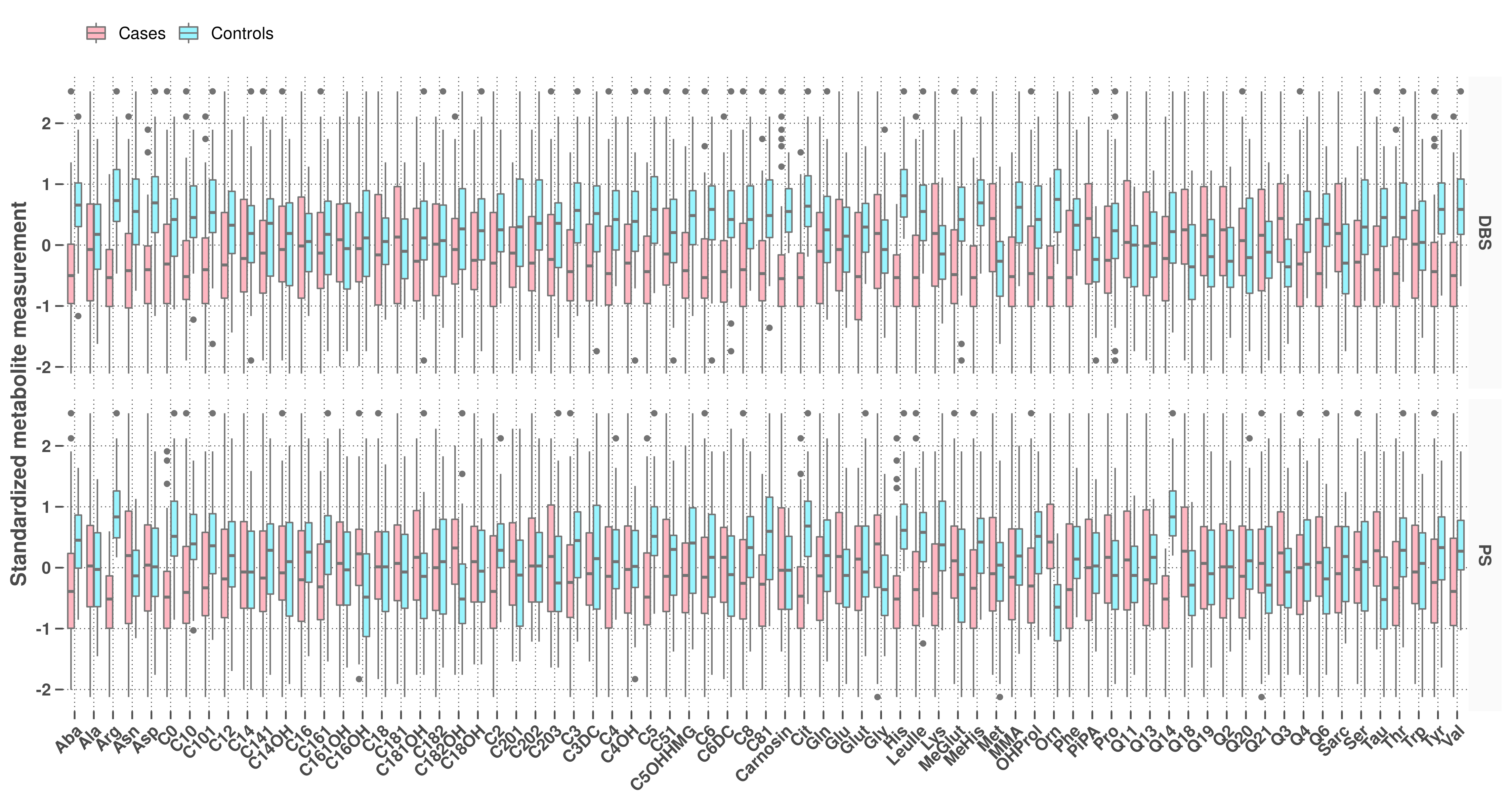

Supplement: Supplementary file 2 — Figure S2. A) Comparison of control standardized metabolite measurements in DBS and PS; B) Comparison of case–control differences among pre‐processed metabolite measurements in plasma and dried blood [file JCSM-12-2101-s005.zip › JCSM_12817_Supplemental Figure S2 B Comparison Pat. vs. Contr.revised.tiff]
