# Supplementary material for: Biomarkers related to fatty acid oxidative capacity are predictive for continued weight loss in cachectic cancer patients
Source: J Cachexia Sarcopenia Muscle. 2021 Oct 11;12(6):2101–10. doi: 10.1002/jcsm.12817 (PMC8718041; doi:10.1002/jcsm.12817)
Supplement: Supplementary file 3 — Table S1. Baseline clinical characteristics and weight variations. Abbreviations: UICC, Union international contre le Cancer; BMI, Body Mass Index [file JCSM-12-2101-s007.docx]

**Supplemental Table S1:** Baseline clinical characteristics and weight variations

Abbreviations: UICC, Union international contre le Cancer; BMI, Body Mass Index;

|  | **Cachexia group** | | |
| --- | --- | --- | --- |
|  | **All**  **(n=18)** | **Weight variations**  **(n=14)** | |
|  |  | **Stable or increase* (n=7)** | **Decrease***  **(n=7)** |
| **Age median (range)** | 61 (51-79) | 67 (51-73) | 63 (52-79) |
| **Gender** |  |  |  |
| male | 14 | 5 | 6 |
| female | 4 | 2 | 1 |
| **BMI (kg/mq)** |  |  |  |
| Underweight (<18.5 kg/mq) | 0 | 0 | 0 |
| Normal weight (18.5 -24.9 kg/mq) | 8 | 3 | 4 |
| Overweight (25.0 - 29.9 kg/mq) | 5 | 2 | 2 |
| Obese Class I-III (>30.0 kg/mq) | 5 | 2 | 1 |
| **Cancer Stage (UICC)** |  |  |  |
| I | - | - | - |
| II | 1 | - | 1 |
| III | 3 | 2 | 1 |
| IV | 14 | 5 | 5 |
| **Tumor site** |  |  |  |
| Gastric | 8 | 3 | 4 |
| Oesophageal | 4 | 1 | 1 |
| Pancreatic | 5 | 2 | 2 |
| Colorectal cancer | 1 | 1 | - |
| **Chemotherapy setting** |  |  |  |
| Neoadjuvant | 3 | 2 | 1 |
| Adjuvant | 1 | - | 1 |
| Palliative | 14 | 5 | 5 |
| **Metabolic disease** |  |  |  |
| Diabetes mellitus | 7 | 5 | 1 |
| Hyperuricemia | 4 | 2 | 2 |
| Hyperlipoproteinemia | 2 | 2 | - |
| Parenteral nutrition at enrollment | 4 | 2 | 1 |

* for all time points
