# Supplementary material for: Biomarkers related to fatty acid oxidative capacity are predictive for continued weight loss in cachectic cancer patients
Source: J Cachexia Sarcopenia Muscle. 2021 Oct 11;12(6):2101–10. doi: 10.1002/jcsm.12817 (PMC8718041; doi:10.1002/jcsm.12817)
Supplement: Supplementary file 4 — Table S2. Spearman Correlation between metabolites in DBS and PS determined in the control cohort [file JCSM-12-2101-s006.docx]

**Supplemental Table S2**: Spearman Correlation between metabolites in DBS and PS determined in the control cohort.

| **Metabolite** | **Spearman’s rho** | **p value** | **q value** |
| --- | --- | --- | --- |
| Pro | 0.86 | <0.0001 | 0.0000 |
| Ala | 0.81 | <0.0001 | 0.0000 |
| Aba | 0.74 | <0.0001 | 0.0000 |
| Phe | 0.72 | <0.0001 | 0.0000 |
| Thr | 0.72 | <0.0001 | 0.0000 |
| C0 | 0.65 | <0.0001 | 0.0002 |
| Q11 | 0.66 | <0.0001 | 0.0002 |
| C10 | 0.65 | <0.0001 | 0.0002 |
| Gly | 0.64 | <0.0001 | 0.0002 |
| Cit | 0.60 | 0.0001 | 0.0007 |
| Leu/Ile | 0.57 | 0.0003 | 0.0019 |
| Tyr | 0.56 | 0.0004 | 0.0025 |
| Sarc | 0.53 | 0.0009 | 0.0052 |
| MeHis | 0.52 | 0.0012 | 0.0065 |
| C4 | 0.50 | 0.0020 | 0.0097 |
| Arg | 0.46 | 0.0043 | 0.0195 |
| C2 | 0.46 | 0.0052 | 0.0224 |
| Q13 | 0.44 | 0.0073 | 0.0296 |
| Ser | 0.43 | 0.0081 | 0.0310 |
| Q6 | 0.43 | 0.0092 | 0.0334 |
| OHProl | 0.40 | 0.0146 | 0.0500 |
| PiPA | 0.40 | 0.0151 | 0.0500 |
| Gln | 0.39 | 0.0183 | 0.0581 |
| C181OH | -0.36 | 0.0291 | 0.0820 |
| His | 0.37 | 0.0275 | 0.0820 |
| Q20 | 0.36 | 0.0292 | 0.0820 |
| Val | 0.35 | 0.0353 | 0.0955 |
| C14OH | -0.34 | 0.0411 | 0.1071 |
| C101 | 0.33 | 0.0501 | 0.1260 |
| C81 | 0.32 | 0.0585 | 0.1423 |
| Q21 | 0.31 | 0.0654 | 0.1540 |
| MMA | 0.30 | 0.0797 | 0.1819 |
| Glut | 0.29 | 0.0825 | 0.1825 |
| Q18 | 0.28 | 0.1040 | 0.2233 |
| Q4 | 0.26 | 0.1211 | 0.2526 |
| Orn | 0.25 | 0.1386 | 0.2810 |
| C3 | 0.23 | 0.1735 | 0.3247 |
| Met | 0.24 | 0.1667 | 0.3247 |
| Q3 | -0.23 | 0.1720 | 0.3247 |
| C18 | 0.20 | 0.2321 | 0.4133 |
| C5OHHMG | 0.21 | 0.2278 | 0.4133 |
| C182OH | 0.18 | 0.2906 | 0.5051 |
| Trp | 0.16 | 0.3373 | 0.5727 |
| MeGlut | 0.16 | 0.3559 | 0.5905 |
| Q2 | -0.15 | 0.3782 | 0.6136 |
| C6 | 0.15 | 0.3898 | 0.6186 |
| C161OH | 0.14 | 0.3999 | 0.6211 |
| C182 | 0.14 | 0.4144 | 0.6303 |
| C12 | 0.13 | 0.4654 | 0.6933 |
| Lys | 0.12 | 0.4751 | 0.6937 |
| Q19 | -0.12 | 0.5021 | 0.7187 |
| C16 | -0.11 | 0.5403 | 0.7307 |
| C18OH | 0.10 | 0.5533 | 0.7307 |
| C3DC | 0.11 | 0.5327 | 0.7307 |
| Carnosin | -0.10 | 0.5605 | 0.7307 |
| Q14 | 0.10 | 0.5481 | 0.7307 |
| C8 | 0.09 | 0.5926 | 0.7590 |
| Asp | 0.08 | 0.6271 | 0.7629 |
| C16OH | -0.09 | 0.6106 | 0.7629 |
| C181 | 0.08 | 0.6238 | 0.7629 |
| C6DC | 0.07 | 0.6973 | 0.8344 |
| Tau | -0.05 | 0.7587 | 0.8933 |
| Asn | 0.04 | 0.8117 | 0.8968 |
| C14 | 0.04 | 0.8209 | 0.8968 |
| C141 | -0.04 | 0.8072 | 0.8968 |
| C201 | 0.04 | 0.8109 | 0.8968 |
| C202 | 0.04 | 0.8231 | 0.8968 |
| C4OH | -0.03 | 0.8512 | 0.9006 |
| Glu | -0.03 | 0.8440 | 0.9006 |
| C203 | -0.03 | 0.8698 | 0.9071 |
| C161 | 0.02 | 0.9209 | 0.9468 |
| C5 | 0.00 | 0.9928 | 0.9928 |
| C51 | 0.00 | 0.9898 | 0.9928 |
