# Supplementary material for: Biomarkers related to fatty acid oxidative capacity are predictive for continued weight loss in cachectic cancer patients
Source: J Cachexia Sarcopenia Muscle. 2021 Oct 11;12(6):2101–10. doi: 10.1002/jcsm.12817 (PMC8718041; doi:10.1002/jcsm.12817)
Supplement: Supplementary file 5 — Table S3. Comparison of estimated mean differences of standardized measurements between patients and controls of amino acids, carnitine, free carnitine, acyl‐carnitines and free fatty acids in PS and corresponding DBS. Negative values correspond to an overall lower mean in the patient group compared to the control group, positive values to a higher mean. A q value<0.05 indicates statistical significance following correction for multiple testing [file JCSM-12-2101-s001.docx]

**Supplemental Table S3:** Comparison of estimated mean differences of standardized measurements between patients and controls of amino acids, carnitine, free carnitine, acyl-carnitines and free fatty acids in PS and corresponding DBS. Negative values correspond to an overall lower mean in the patient group compared to the control group, positive values to a higher mean. A q value<0.05 indicates statistical significance following correction for multiple testing.

|  | **DBS** | | | **Plasma** | | |
| --- | --- | --- | --- | --- | --- | --- |
|  | **mean difference** | **95% C.I.** | **q value** | **mean difference** | **95% C.I.** | **q value** |
| Aba | -0.508 | -0.974 – -0.0413 | 0.1262 | -0.0609 | -0.547 – 0.425 | 0.9356 |
| Ala | -0.285 | -0.793 – 0.223 | 0.4843 | -0.264 | -0.754 – 0.227 | 0.4907 |
| Asp | -0.766 | -1.41 – -0.117 | 0.0908 | 0.225 | -0.378 – 0.827 | 0.6231 |
| C0 | -0.128 | -0.806 – 0.551 | 0.8192 | -0.441 | -0.967 – 0.0855 | 0.4089 |
| C10 | -0.316 | -0.956 – 0.325 | 0.5546 | 0.00433 | -0.664 – 0.673 | 0.9918 |
| C10:1 | -0.612 | -1.13 – -0.0946 | 0.0908 | -0.299 | -0.787 – 0.19 | 0.4907 |
| C12 | 0.0372 | -0.485 – 0.56 | 0.9126 | 0.0207 | -0.473 – 0.514 | 0.9846 |
| C14 | 0.0215 | -0.583 – 0.626 | 0.9411 | 0.698 | 0.14 – 1.26 | 0.1258 |
| C14;1 | 0.0782 | -0.6 – 0.757 | 0.8941 | 0.362 | -0.271 – 0.994 | 0.4907 |
| C14OH | 0.345 | -0.189 – 0.879 | 0.3997 | 0.0367 | -0.463 – 0.537 | 0.9636 |
| C16 | 0.168 | -0.618 – 0.954 | 0.8103 | 0.00285 | -0.577 – 0.583 | 0.9918 |
| C16:1OH | 0.169 | -0.396 – 0.734 | 0.7297 | 0.0458 | -0.68–0.772 | 0.9636 |
| C16OH | 0.133 | -0.315 – 0.582 | 0.7297 | 0.529 | 0.108 – 0.951 | 0.1258 |
| C18 | 0.194 | -0.536 – 0.924 | 0.7436 | 0.397 | -0.202 – 0.997 | 0.4907 |
| C18:1 | 0.432 | -0.24 – 1.1 | 0.3997 | 0.36 | -0.121 – 0.841 | 0.4740 |
| C18:1OH | 0.368 | -0.15 – 0.886 | 0.3465 | 0.34 | -0.268 – 0.948 | 0.4907 |
| C18:2 | 0.0689 | -0.567 – 0.705 | 0.8941 | 0.0403 | -0.483 – 0.564 | 0.9636 |
| C18:2OH | -0.266 | -0.862 – 0.331 | 0.5854 | 0.511 | -0.0373 – 1.06 | 0.3431 |
| C18OH | -0.0624 | -0.679 – 0.554 | 0.8941 | 0.142 | -0.45 – 0.735 | 0.7921 |
| C2 | -0.173 | -0.982 – 0.636 | 0.8103 | 0.192 | -0.405 – 0.79 | 0.6598 |
| C20:1 | -0.241 | -0.759 – 0.276 | 0.5736 | 0.336 | -0.281 – 0.954 | 0.4907 |
| C20:2 | -0.475 | -1.14 – 0.194 | 0.3465 | -0.247 | -0.645 – 0.151 | 0.4907 |
| C20:3 | 0.161 | -0.439 – 0.761 | 0.7436 | 0.231 | -0.295 – 0.757 | 0.5948 |
| C3 | -0.533 | -1.08 – 0.01 | 0.1573 | -0.0512 | -0.526 – 0.423 | 0.9531 |
| C3DC | -0.597 | -1.08 – -0.114 | 0.0852 | -0.299 | -0.796 – 0.199 | 0.4907 |
| C4 | -0.294 | -0.957 – 0.369 | 0.5854 | 0.043 | -0.651 – 0.736 | 0.9636 |
| C4OH | 0.272 | -0.354 – 0.899 | 0.5879 | 0.446 | -0.295 – 1.19 | 0.4907 |
| C5 | -0.388 | -1.04 – 0.268 | 0.4489 | -0.703 | -1.32 – -0.0868 | 0.1665 |
| C5:1 | 0.151 | -0.345 – 0.647 | 0.7297 | -0.00864 | -0.621 – 0.603 | 0.9918 |
| C5OH+HMG | -0.117 | -0.68 – 0.446 | 0.8103 | -0.2 | -0.767 – 0.367 | 0.6231 |
| C6DC | -0.184 | -0.863 – 0.496 | 0.7436 | 0.436 | -0.194 – 1.07 | 0.4740 |
| C8 | -0.24 | -0.83 – 0.35 | 0.6237 | -0.162 | -0.862–0.538 | 0.7939 |
| Glu | -0.253 | -0.991 – 0.485 | 0.7278 | 0.312 | -0.37 – 0.994 | 0.5788 |
| Glut | 0.154 | -0.326 – 0.635 | 0.7297 | 0.247 | -0.447 – 0.94 | 0.6231 |
| Gly | -0.537 | -1.06 – -0.0115 | 0.1391 | -0.239 | -0.804 – 0.325 | 0.5954 |
| Lys | 0.696 | 0.118 – 1.27 | 0.0908 | -0.497 | -1.07 – 0.0752 | 0.3885 |
| MMA | -0.783 | -1.31 – -0.255 | 0.0420 | 0.104 | -0.601 – 0.808 | 0.9098 |
| MeGlut | -0.0784 | -0.715 – 0.558 | 0.8941 | 0.417 | -0.177 – 1.01 | 0.4740 |
| OH-Prol | -0.322 | -0.842 – 0.198 | 0.4217 | -0.413 | -0.939 – 0.113 | 0.4713 |
| Phe | -0.603 | -1.19 – -0.016 | 0.1391 | -0.288 | -0.965 – 0.39 | 0.5954 |
| PiPA | 0.0897 | -0.618 – 0.798 | 0.8941 | -0.484 | -1.17 – 0.199 | 0.4740 |
| Pro | -0.31 | -0.974 – 0.354 | 0.5736 | 0.332 | -0.234 – 0.897 | 0.4907 |
| Q11 | 0.0644 | -0.668 – 0.797 | 0.9041 | -0.39 | -0.95 – 0.171 | 0.4740 |
| Q13 | 0.0451 | -0.621 – 0.711 | 0.9126 | -0.00991 | -0.572 – 0.552 | 0.9918 |
| Q19 | 0.0955 | -0.527 – 0.718 | 0.8680 | -0.304 | -0.736 – 0.127 | 0.4740 |
| Q2 | 0.4 | -0.327 – 1.13 | 0.4874 | -0.169 | -0.595 – 0.258 | 0.6192 |
| Q20 | 0.28 | -0.247 – 0.807 | 0.5041 | -0.332 | -0.897 – 0.233 | 0.4907 |
| Q21 | 0.653 | 0.018 – 1.29 | 0.1391 | 0.232 | -0.43 – 0.894 | 0.6231 |
| Q3 | 0.64 | -0.0436 – 1.32 | 0.1814 | 0.0435 | -0.405 – 0.492 | 0.9585 |
| Q4 | -0.188 | -0.824 – 0.448 | 0.7297 | 0.0663 | -0.36 – 0.492 | 0.9083 |
| Q6 | -0.513 | -1.1 – 0.0729 | 0.2136 | 0.381 | -0.322 – 1.08 | 0.4907 |
| Sarc | -0.0234 | -0.684 – 0.637 | 0.9411 | -0.304 | -0.804 – 0.197 | 0.4907 |
| Tau | -0.589 | -1.33 – 0.156 | 0.2851 | 0.658 | 0.0732 – 1.24 | 0.1665 |
| Trp | -0.177 | -0.727 – 0.372 | 0.7297 | 0.263 | -0.447 – 0.973 | 0.6231 |
| Tyr | -0.644 | -1.24 – -0.0465 | 0.1262 | -0.359 | -1.03 – 0.314 | 0.4907 |
| Val | -0.686 | -1.22 – -0.154 | 0.0713 | -0.266 | -0.995 – 0.463 | 0.6231 |

Abbreviations: DBS. dried blood sample; 95% C.I. 95% Confidence Interval; abbreviations of analytes and ratios see supplemental table S5;
