# Supplementary material for: Biomarkers related to fatty acid oxidative capacity are predictive for continued weight loss in cachectic cancer patients
Source: J Cachexia Sarcopenia Muscle. 2021 Oct 11;12(6):2101–10. doi: 10.1002/jcsm.12817 (PMC8718041; doi:10.1002/jcsm.12817)
Supplement: Supplementary file 6 — Table S4. A) Linear regression analysis of metabolome profile predictive for initial weight loss as a continuous variable (according to numeric weight loss from the historical weight to the weight at TP1); B) Linear regression analysis of metabolome profile (according to numeric weight loss) from baseline to the second scheduled evaluation (i.e., early weight loss). Only results with p‐value <0.05 are reported, q‐values represent results following correction for multiple testing [file JCSM-12-2101-s002.docx]

**Supplemental Table S4:** A) Linear regression analysis of metabolome profile predictive for initial weight loss as a continuous variable (according to numeric weight loss from the historical weight to the weight at TP1); B) Linear regression analysis of metabolome profile (according to numeric weight loss) from baseline to the second scheduled evaluation (i.e., early weight loss). Only results with p-value <0.05 are reported, q-values represent results following correction for multiple testing.

| **A)** | **Tissue** | **Beta** | **Beta SE** | **T statistic** | **p value** | **q value** |
| --- | --- | --- | --- | --- | --- | --- |
| C14 | DBS | -0.0381842 | 0.0164235 | -2.324973 | 0.040 | 0.741 |
| C20:2 | Plasma | 0.0497061 | 0.0204190 | 2.434302 | 0.032 | 0.971 |
| Leu/Ileu | DBS | -0.0504618 | 0.0205612 | -2.454226 | 0.032 | 0.741 |
| OHProl | DBS | -0.0409679 | 0.0176645 | -2.319219 | 0.041 | 0.742 |
| Orn | Plasma | 0.0568179 | 0.0205865 | 2.759961 | 0.017 | 0.971 |
| Trp | DBS | 0.0428139 | 0.0181645 | 2.357014 | 0.038 | 0.742 |
| **B)** | **Tissue** | **Beta** | **Beta SE** | **T statistic** | **p value** | **q value** |
| Ala | DBS | -3.930078 | 1.316661 | -2.984882 | 0.0124 | 0.181 |
| C201 | DBS | 4.170614 | 1.139608 | 3.659693 | 0.0037 | 0.149 |
| Q14 | DBS | 3.604727 | 1.166638 | 3.089842 | 0.0103 | 0.181 |
| Q19 | Plasma | 3.617000 | 1.430785 | 2.527982 | 0.0265 | 0.918 |
| Q2 | DBS | 3.419469 | 1.003448 | 3.407719 | 0.0058 | 0.149 |
| Q3 | DBS | 3.721113 | 1.101238 | 3.379027 | 0.006 | 0.149 |

Abbreviations: DBS, dried blood sample; 95% C.I. 95% Confidence Interval; abbreviations of analytes and ratios are given in supplemental table S5.
