# Supplementary material for: Biomarkers related to fatty acid oxidative capacity are predictive for continued weight loss in cachectic cancer patients
Source: J Cachexia Sarcopenia Muscle. 2021 Oct 11;12(6):2101–10. doi: 10.1002/jcsm.12817 (PMC8718041; doi:10.1002/jcsm.12817)
Supplement: Supplementary file 7 — Table S5. Complete overview of metabolites and metabolite ratios analyzed and mapped in the reference metabolite database as published by Burkhardt et al. [15]. HMDB and PubChem identifiers are indicated [file JCSM-12-2101-s004.docx]

**Supplemental Table S5:** Complete overview of metabolites and metabolite ratios analyzed and mapped in the reference metabolite database as published by Burkhardt et al.[40]

| **Abbreviation** | **Full Name** | **Pathway** | **Superpathway** | **HMDB** | **PubChem** |
| --- | --- | --- | --- | --- | --- |
| Aba | Aminobutyric acid | GABA, AABA | Other | HMDB0000112 | 119 |
| AC-total | Acylcarnitine total | Carnitine transport | Carnitine transport | - | - |
| Ala | Alanine | Muscle metabolism, Ammonia-carrier, Glucose metabolism | Urea cycle | HMDB0000161 | 5950 |
| Arg | Arginine | Urea cycle, NO metabolism | Urea cycle | HMDB0000517 | 6322 |
| Asp | Aspartic acid | Amino group recycling | Ammonia recycling | HMDB0000191 | 5960 |
| C0 | Carnitine free | Carnitine transport | Carnitine transport | HMDB0000062 | 2724480 |
| C10 | Decanoylcarnitine | Medium chain fatty acid metabolism | Fatty acid metabolism | HMDB0000651 | 10245190 |
| C10:1 | Decenoylcarnitine | Medium chain fatty acid metabolism | Fatty acid metabolism | HMDB0013205 | 53481651 |
| C12 | Dodecanoylcarnitine | Medium chain fatty acid metabolism | Fatty acid metabolism | HMDB0002250 | 168381 |
| C14 | Myristoylcarnitine | Long chain fatty acid metabolism | Fatty acid metabolism | HMDB0005066 | 53477791 |
| C14:1 | Tetradecenoylcarnitine | Long chain fatty acid metabolism | Fatty acid metabolism | HMDB02014/HMDB0013329 | 22833575/53481677 |
| C14OH | 3-Hydroxy-tetradecanoylcarnitine | Long chain fatty acid metabolism | Fatty acid metabolism | HMDB0061640 | 71464541 |
| C16 | Palmitoylcarnitine | Long chain fatty acid metabolism | Fatty acid metabolism | HMDB0000222 | 11953816 |
| C16:1 | Hexadecenoylcarnitine | Long chain fatty acid metabolism | Fatty acid metabolism | HMDB0013207 | 53481653 |
| C16:1OH | 3-Hydroxy-hexadecenoylcarnitine | Long chain fatty acid metabolism | Fatty acid metabolism | HMDB0013333 | 53481685 |
| C16OH | 3-Hydroxy-hexadecanoylcarnitine | Long chain fatty acid metabolism | Fatty acid metabolism | HMDB0013336 | 53481691 |
| C18 | Stearoylcarnitine | Long chain fatty acid metabolism | Fatty acid metabolism | HMDB0000848 | 6426855 |
| C18:1 | Octadecenoylcarnitine | Long chain fatty acid metabolism | Fatty acid metabolism | HMDB0094687 | 87076413 |
| C18:1OH | Hydroxy-octadec-1-enoylcarnitine | Long chain fatty acid metabolism | Fatty acid metabolism | HMDB0013339 | 53481697 |
| C18:2 | Trans, trans-9,12-octadecadienoic acid (Linoelaidic) | Long chain fatty acid metaolism | Fatty acid metabolism | HMDB0006469/HMDB0006461 | 6450015/53477834 |
| C18:2OH | Hydroxy-octadec-2-enoylcarnitine | Long chain fatty acid metabolism | Fatty acid metabolism | - | 71464556 |
| C18OH | 3-Hydroxy-octadecanoylcarnitine | Long chain fatty acid metabolism | Fatty acid metabolism | HMDB0013154 | 53481632 |
| C2 | Acetylcarnitine | Energy metabolism | Energy metabolism | HMDB0000201 | 7045767 |
| C20:1 | Cis-11-eicosenoic acid | PUFA metabolism | Fatty acid metabolism | HMDB0002231 | 5282768 |
| C20:2 | Cis-11,14-eicosadienoic acid | PUFA metabolism | Fatty acid metabolism | HMDB0005060 | 6439848 |
| C20:3 | Cis-11,14,17-eicosatrienoic acid methyl ester | PUFA metabolism | Fatty acid metabolism | - | 77174731 |
| C3 | Propionylcarnitine | Isoleucine metabolism, Valine metabolism | BCAA metabolism | HMDB00824 | 107738 |
| C3DC | Malonylcarnitine | Energy metabolism, Fatty acid synthesis | BCAA metabolism | HMDB02095 | 22833583 |
| C4 | Butyrylcarnitine | Short chain fatty acid metabolism | Energy metabolism | HMDB02013 | 439829 |
| C4OH | 3-Hydroxy-butyryl-carnitine | Energy metabolism | Energy metabolism | HMDB0013127 | 71464477 |
| C5 | Isovalerylcarnitine | Leucine metabolism | BCAA metabolism | HMDB0000688 | 6426851 |
| C5:1 | Triglylcarnitine | Isoleucine metabolism, Valine metabolism | BCAA metabolism | HMDB0002366 | 91825636 |
| C5OH+HMG | 2-Hydroxyisovalerylcarnitine | Leucine metabolism | BCAA metabolism | HMDB0062555 | 57357187 |
| C6 | Hexanoylcarnitine | Medium chain fatty acid metabolism | Fatty acid metabolism | HMDB0000705 | 6426853 |
| C6DC | Adipylcarnitine (3-Methylglutarylcarnitine) | Keton body generation from beta oxidation and ketogenic amino acids | Fatty acid metabolism | HMDB0061677 | 71296139 |
| C8 | Octanoylcarnitine | Medium chain fatty acid metabolism | Fatty acid metabolism | HMDB00791 | 11953814 |
| C8:1 | Octenoylcarnitine | Medium chain fatty acid metabolism | Fatty acid metabolism | - | 71464472 |
| Carn | Carnosin | Histidine metabolism, Beta-alanine metabolism | Amino acid metabolism, other | HMDB0000033 | 439224 |
| Cit | Citrulline | Urea cycle, NO metabolism | Urea cycle | HMDB0000904 | 9750 |
| Gln | Glutamine | Amino group recycling | Ammonia recycling | HMDB0000641 | 5961 |
| Glu | Glutamic acid | Amino group recycling, GABA production | Ammonia recycling | HMDB0000148 | 33032 |
| Glut | Glutarylcarnitine | Lysine metabolism, Thryptophan metabolism, Beta-oxidation | Amino acid metabolism, other | HMDB0013130 | 71317118 |
| Gly | Glycine | Serin katabolism, brain function, purine synthesis, porphyrin synthesis | Ammonia recycling | HMDB0000123 | 750 |
| His | Histidine | Histamine precorsor, Carnosin precursor | Amino acid metabolism, other | HMDB0000177 | 6274 |
| Leu \| Ile | Leucine \| Isoleucine | Leucine metabolsim, Isoleucine metabolism | BCAA metabolism | HMDB00687/HMDB0000172 | 6106/6306 |
| Lys | Lysine | Carnitine precursor, collagen, glutamate generation | Amino acid metabolism, other | HMDB0000182 | 5962 |
| MeGlut | 3-Methylglutarylcarnitine | Leucine metabolism | Amino acid metabolism, other | HMDB0000552 | 128145 |
| MeHis | Methyl-histidine | Histidine metabolism | Amino acid metabolism, other | HMDB0000001 | 92105 |
| Met | Methionine | Cytosolic methyl group transfer, intermediate in the biosynthesis of cysteine, carnitine, taurine, lecithin, phosphatidylcholine, and other phospholipids | Amino acid metabolism, other | HMDB0000696 | 6137 |
| MMA | Methylmalonylcarnitine | Valine metabolism, Vitamine B12 metabolism | BCAA metabolism | HMDB0013133 | 53481628 |
| OH-Prol | Hydroxyproline | Collagen synthesis | Collagen synthesis | HMDB0000725 | 5810 |
| Orn | Ornithine | Urea cycle | Urea cycle | HMDB0000214 | 6262 |
| Phe | Phenylalanine | Dopamine precursor | Amino acid metabolism, other | HMDB0000159 | 6140 |
| PiPA | Pipecolic acid | Peroxisomal metabolism, Lysine degradation | Amino acid metabolism, other | HMDB0000070 | 849 |
| Pro | Proline | Collagen synthesis | Collagen synthesis | HMDB0000162 | 145742 |
| Sarc | Sarcosine | Glycine katabolism | Amino acid metabolism, other | HMDB0000271 | 1088 |
| Ser | Serine | Glycine formation | Ammonia recycling | HMDB0000187 | 5951 |
| Tau | Taurine | Cystein metabolism, bile acid generation | Amino acid metabolism, other | HMDB0000251 | 1123 |
| Thr | Threonine | Glycine precursor, Serine precursor | Amino acid metabolism, other | HMDB0000167 | 6288 |
| Trp | Tryptophan | Serotonine precursor | Amino acid metabolism, other | HMDB0000929 | 6305 |
| Tyr | Tyrosine | Dopamine precursor | Amino acid metabolism, other | HMDB0000158 | 6057 |
| Val | Valine | Valine metabolism | BCAA metabolism | HMDB0000883 | 6287 |
| Aba | Aminobutyric acid | GABA, AABA | Other | HMDB0000112 | 119 |
| **Description of Metabolite Ratios** | | | |  |  |
| **Abbreviation** | **Full name** | **Pathway** | **Superpathway** |  |  |
| Q1:(Val+Leu\|Ile)/(Phe+Tyr) | (Valine + Leucine\|Isoleucine) / (Phenylalanine + Tyrosine) | Phenylalanine and tyrosine metabolism | BCAA metabolism |  |  |
| Q2:C16/C2 | Palmitoylcarnitine / Acetylcarnitine | CPT I, CPT II, Beta-oxidation | Energy metabolism |  |  |
| Q3:(C16+C18:1)/C2 | (Palmitoylcarnitine + Octadecenoylcarnitine) / Acetylcarnitine | CPT I, CPT II, Beta-oxidation | Energy metabolism |  |  |
| Q4:C0/(C16+C18) | Carnitine free / Palmitoylcarnitine + Stearoylcarnitine | Carnitine transport | Energy metabolism |  |  |
| Q5:Gln/Glu | Glutamine / Glutamic acid | Glutamate precursor of glutamine | Ammonia recycling |  |  |
| Q6:Glut/Lys | Glutarylcarnitine / Lysine | Lysine metabolism, Beta-oxidation | Amino acid metabolism, other |  |  |
| Q8:Pro/(OH-Prol) | Proline / Hydroxyproline | Collagen synthesis | Collagen synthesis |  |  |
| Q9:Lys/PiPA | Lysine / Pipecolic acid | Lysine degradation | Amino acid metabolism, other |  |  |
| Q11:Ala/C2 | Alanine / Acetylcarnitine | Glucose metabolism | Energy metabolism |  |  |
| Q12:Ala/Asp | Alanine / Aspartic acid | Muscle energy, Glucose metabolism | Energy metabolism |  |  |
| Q13:Arg/Cit | Arginine / Citrulline | Urea cycle | Urea cycle |  |  |
| Q14:Arg/Orn | Arginine / Ornithine | Urea cycle | Urea cycle |  |  |
| Q15:Arg/Gly | Arginine / Glycine | Ammonia recycling | Urea cycle |  |  |
| Q16:Asp/Cit | Aspartic acid / Citrulline | Ammonia recycling | Ammonia recycling |  |  |
| Q17:Glu/Pro | Glutamic acid / Proline | Glutamate precursor of proline biosynthesis | Collagen synthesis |  |  |
| Q18: C5:1/(Leu\|Ile) | Tiglylcarnitine / Leucine\|Isoleucine | Isoleucine metabolism | BCAA metabolism |  |  |
| Q19:(Leu\|Ile)/C3 | Leucine\|Isoleucine / Propionylcarnitine | Isoleucine metabolism, Valine metabolism | BCAA metabolism |  |  |
| Q20:C5/(Leu\|Ile) | Isovalerylcarnitine / Leucine\|Isoleucine | Leucine metabolism | BCAA metabolism |  |  |
| Q21:(C5OH+HMG)/(Leu\|Ile) | 2-Hydroxyisovalerylcarnitine / Leucine\|Isoleucine | Leucine metabolism | BCAA metabolism |  |  |
| Q22:Met/Ser | Methionine / Serine | Folic acid cycle | Folic acid cycle |  |  |
| Q23:Met/Gly | Methionine / Glycine | Cytosolic methyl group transfer | Amino acid metabolism, other |  |  |
| Q24:Met/Tau | Methionine / Taurine | Taurine synthesis | Amino acid metabolism, other |  |  |
| Q25:Pro/Orn | Proline / Ornithine | Glutamate precursor of ornithine biosynthesis | Collagen synthesis |  |  |
| Q26:Glu/Orn | Glutamic acid / Ornithine | Ammonia recycling | Ammonia recycling |  |  |
| Q27:Sarc/Gly | Sarcosine / Glycine | Glycine metabolism | Amino acid metabolism, other |  |  |
| Q28:Ser/Gly | Serine / Glycine | Folic acid cycle | Folic acid cycle |  |  |
| Q30:Phe/Tyr | Phenylalanine / Tyrosine | Phenylalanine and tyrosine metabolism | Amino acid metabolism, other |  |  |
| Q32:C4OH/Val | 3-Hydroxy-(iso)butyryl-carnitine / Valine | Valin metabolism | BCAA metabolism |  |  |
| Q33:C0/(AC-total) | Carnitine free / Acylcarnitine total | Carnitine transport, Beta-oxidation | Energy metabolism |  |  |
| Q34:Asp/C2 | Aspartic acid / Acetylcarnitine | Ammonia recycling | Ammonia recycling |  |  |
| Q35:C5/C4 | Isovalerylcarnitine / Butyrylcarnitine | Leucine metabolism | Energy metabolism |  |  |
| Q36:C8/C10 | Octanoylcarnitine / Decanoylcarnitine | MCAD, Beta-oxidation | Energy metabolism |  |  |
| Q37:(C14:1)/C14 | Tetradecenoylcarnitine / Myristoylcarnitine | VLCAD, Beta-oxidation | Energy metabolism |  |  |
| Q38:MMA/C3 | Methylmalonylcarnitine / Propionylcarnitine | Isoleucine metabolism, Valine metabolism | BCAA metabolism |  |  |
